# Clinical Assessment of Immediate Autotransplantation of Mandibular Third Molars: An In Vivo Study

**DOI:** 10.7759/cureus.41293

**Published:** 2023-07-02

**Authors:** Channaveer Pattanshetti, Banashree Sankeshwari, Santaji Shinde, Poornima Kadam, Harshawardhan Kadam, Amol Shirkande

**Affiliations:** 1 Oral and Maxillofacial Surgery, Bharati Vidyapeeth Dental College and Hospital, Sangli, IND; 2 Prosthodontics, Bharati Vidyapeeth Dental College and Hospital, Sangli, IND; 3 Pathology, Bharati Vidyapeeth Medical College and Hospital, Sangli, IND; 4 Orthodontics and Dentofacial Orthopaedics, Bharati Vidyapeeth Dental College and Hospital, Sangli, IND

**Keywords:** intentional reimplantation, impacted tooth, tooth transplantation, autogenous transplantation, success rate

## Abstract

Introduction: Transplanting a tooth from one area of the mouth to another is known as autogenous tooth transplantation. It is a great choice for restoring young patients' teeth with developing alveolar bone because it uses the patient's own tooth as the replacement rather than a false one. This study aims to evaluate pain, infection, mobility, resorption, ankylosis, and success rate in the replacement of mandibular non-restorable molars through an immediate autotransplantation of the nonfunctional impacted mandibular third molar.

Materials and methods: In this in vivo study, 20 patients between the ages of 22 and 50 were selected. The cases in which the first or second mandibular molar was nonrestorable and had an impacted third molar for transplantation were selected. In all the cases, the nonrestorable molar was extracted and replaced with a nonfunctional, impacted third molar. All the cases were evaluated for pain, infection, mobility, ankylosis, and resorption at the postoperative second week, one month, third month, and six months. The pain was assessed on the visual analog scale (VAS), infection was assessed by the presence of purulent discharges, mobility was assessed on a clinical examination of tooth movements, ankylosis was seen radiographically as the obliteration of the periodontal ligament space, and the absence of the lamina dura and resorption were seen radiographically as radiolucency on the root surface. All the readings were tabulated and statistically analyzed.

Results: Pain was seen to be maximal at two weeks and minimum at six months. Infection was not seen at all time intervals. Mobility was reduced with time. There was no significant ankylosis or resorption. Out of the 20 cases, four patients required extractions due to resorption and grade 3 mobility. One patient showed ankylosis. The success rate of the autotransplantation was 75%. Fifteen patients showed well-defined lamina dura without ankylosis or resorption.

Conclusion: Autotransplantation is a valuable tooth replacement option and more economical, especially when provided with proper case selection.

## Introduction

Abulcasis was the first to report autotransplantation in 1050; nevertheless, the first recorded operation describing tooth bud transplantation did not occur until 1564, when it was conducted by a French dentist named Ambroise Paré. John Hunter, a pioneer in the field of dental transplantation and reimplantation, first demonstrated the tooth-to-tissue bonding properties of a human tooth hetero-transplanted into a cock's crest in 1771 [1].

There are three categories that may be used to describe tooth transplantation: autogenous, homogeneous, and heterogeneous. A tooth or tooth bud can be transplanted in three different ways: autogenously, when one person's tooth or bud is placed into another person's socket; homogeneously, when one species' tooth or bud is placed into another species' socket; and heterogeneously, when one species' tooth or bud is placed into another species' socket [2]. When it comes to mandibular molars, early tooth loss due to dental caries is the most frequent ailment and a challenge for dentists. The first molars appear early in life and need extensive restoration. Extraction alone and extraction followed by replacement with an implant-supported crown, fixed dental prosthesis, or removable dental prosthesis are only two ways to deal with premature tooth loss. Autotransplanted teeth, in contrast to dental inserts, function exactly like natural teeth [3,4]. The periodontal ligament (PDL), bone, and soft tissue may continue to grow at the recipient location [5], so this method can also be used to move the teeth with braces after autotransplantation [6,7]. Orthodontic pressure has been applied over a period of four to eight weeks in autotransplanted teeth [8]. Improved aesthetics, dentofacial growth, proprioception, and dental arch integrity are some of the most notable outcomes of this method [9].

Tooth agenesis, especially of the premolars and lateral incisors, traumatic tooth loss, atopic eruption of canines, root resorption, extensive endodontic lesions, cervical root fractures, and localized juvenile periodontitis are some of the other disorders for which transplantation may be a possibility [10-12]. Regional odontodysplasia, tooth aplasia, and cleidocranial dysplasia, which are dental development abnormalities [13,14], and tooth agenesis [15] are a few examples of when transplants may be necessary. With the surge in replacing lost teeth with implants, even though autotransplantation is considered a viable treatment option, it is not commonly followed. Although the autotransplantation of front teeth with full root creation is occasionally done for orthodontic purposes, the practice is far less common for posterior teeth. Autotransplantation of posterior teeth was evaluated in research, with premolar transplants showing higher success rates than molar transplants [16].

There are very few studies that have studied the success rate of impacted mandibular molar autotransplantation [2]. Hence, the aim of this in vivo study is to evaluate the success rate of mandibular impacted third molar autotransplantation in place of mandibular first or second molars.

## Materials and methods

This research is a cross-sectional analysis of real-life samples. Twenty cases were chosen from those reported to the Division of Oral and Maxillofacial Surgery. The patients' ages ranged from 22 to 50, with a mean of 36 years old, seven men (35%) and 13 women (65%). The research was conducted over the course of 112 years (from September 2016 to March 2018) at Bharati Vidyapeeth Dental College and Hospital in Sangli, India. Ethical clearance was obtained to carry out the study (BVDCMC&H/Sangli/IEC/Dissertation 2015-16/160).

The inclusion criteria include patients with an age group of 22-50 years, male and female individuals; patients having an impacted mandibular third molar that can be extracted without sectioning; non-restorable mandibular first or second molars having approximately the same or more size; the size of donor and recipient molars being approximately the same; donor tooth having complete root formation; and physically and mentally fit patients without any known source of infection in the oral cavity. Simple mesioangular impacted molars that can be removed without splitting are selected. The exclusion criteria included patients with systemic diseases (e.g., diabetes, hypertension, bleeding disorders, and kidney and liver diseases), donor teeth with incomplete root formation, and unwillingness or inability to be followed up for radiographic and clinical examination.

Twenty individuals were chosen for this in vivo cross-sectional investigation, It was a time-bound study done for 1.5 years. The cases reported in this tenure were selected and randomly divided, all of whom had impacted third mandibular molars and were missing either their first or second permanent molars. The patient's medical history was well documented, and their informed consent was obtained prior to any surgical procedures. Regular blood tests were required for the patients. All procedures were performed by the same operational surgeon using the same equipment and local anesthetic (2% lignocaine with adrenaline). Before the surgery, panoramic X-rays were taken to ensure that all third molars were in the correct position and had fully developed roots. All patients were set up for surgery in accordance with standard operating procedures. A 2% lignocaine hydrochloride solution with a 1:80,000 adrenaline solution was used to provide a pterygomandibular block. A tooth that could not be saved was removed. In all the cases, the inter-radicular bone had to be removed from the recipient site, and more bone had to be removed from beyond the apex to provide a tension-free apical cushion. To prevent early occlusal interactions, the transplant was placed at or slightly below the occlusal plane owing to the apical preparation.

**Figure 1:**
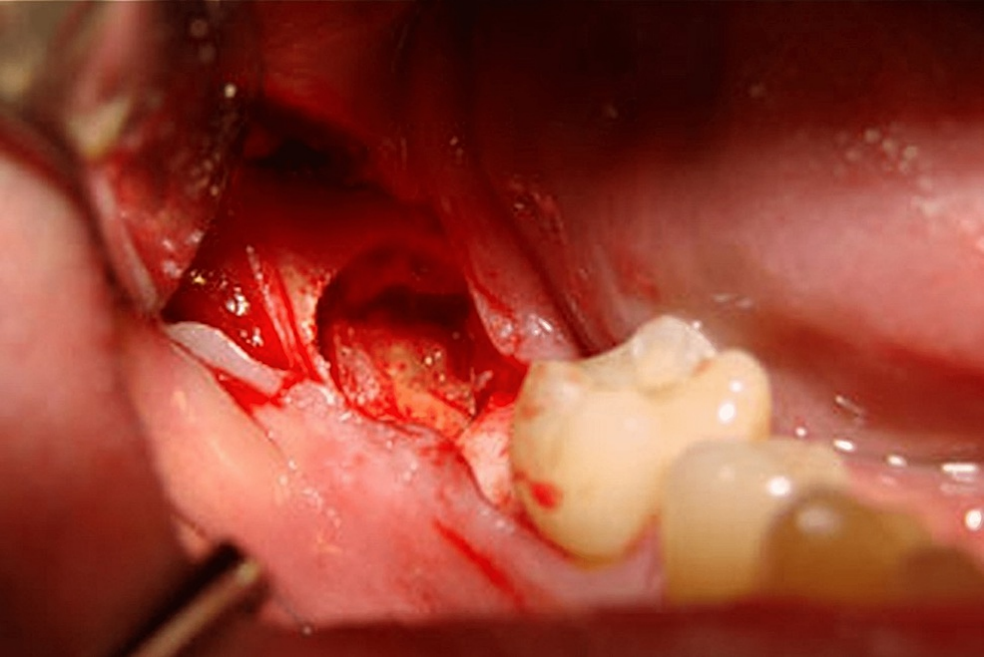
Extraction socket

A full-thickness mucoperiosteal flap was mirrored via a Ward's incision. The incision started below the buccal sulcus. An incision was performed at the gingival border of the upper right second molar, starting at the distobuccal line angle. The incision was then made from the cervical region behind the second molar to the distal midpoint of the tooth. From there, a rearward buccal incision was made along the exterior oblique ridge. The mucoperiosteal flap's full thickness was mirrored using a periosteal elevator (Figure 1).

**Figure 2:**
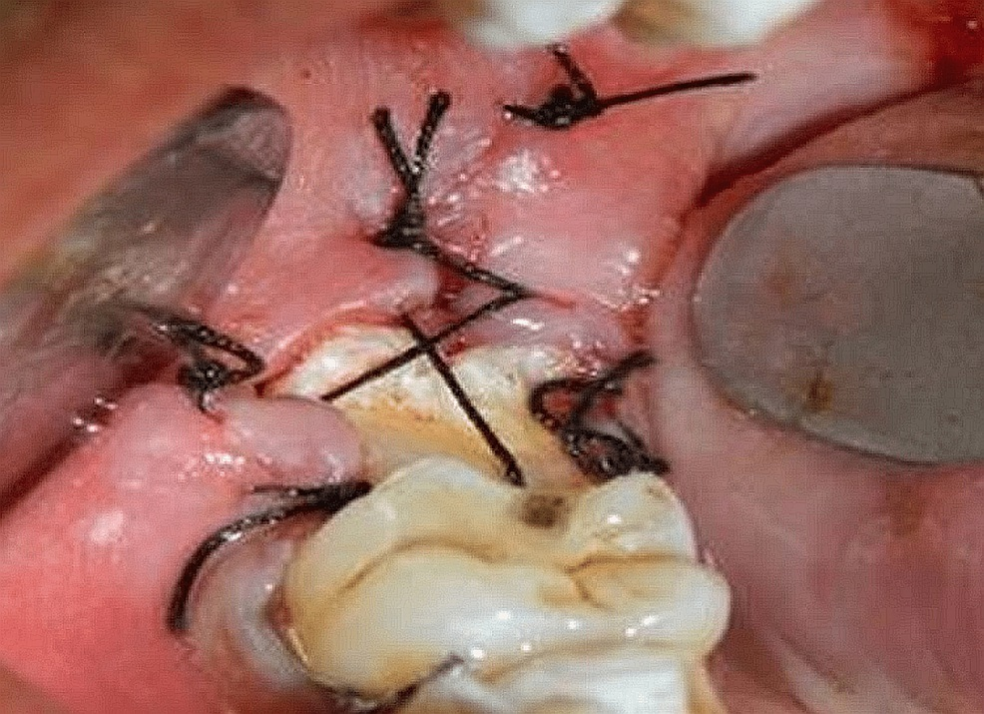
Tooth stabilized after transplantation

Using a straight elevator, the impacted third molar was loosened before being removed using molar forceps and very little pressure. To flush out the incision and eliminate any remaining debris, a sterile saline solution was gently injected into the area. The tissue edges were given a new lease on life. After the impacted tooth was extracted using the routing minor surgical kit, the prepared socket of the non-restorable tooth was used to place the extracted tooth. If the recipient site needed to be further prepared after the third molar was transferred from the donor site, a bone trimmer was used to prepare the socket for reimplantation. It was placed between two gauze pads saturated with a normal saline solution. It was mandated that you take extra care not to touch the root sheath or any of the surgical tools. In cases where the transplanted molar was at risk of making a premature occlusal contact, the recipient site was further prepared. Positions 1-2 mm below the occlusal plane were regarded as optimal, but no occlusal modifications were made. No. 3 silk sutures or composite strips were used to stabilize the transplanted third molar and prevent it from moving vertically. The reimplanted tooth was splinted to an adjacent tooth with an 18-gauge wire and composite. The donor's tooth had its gingival flap securely closed over it. Sutures were used to seal the extraction socket of the donated tooth. When initial stability was not attained, only then did we resort to wire fixing (Figure 2).

**Figure 3:**
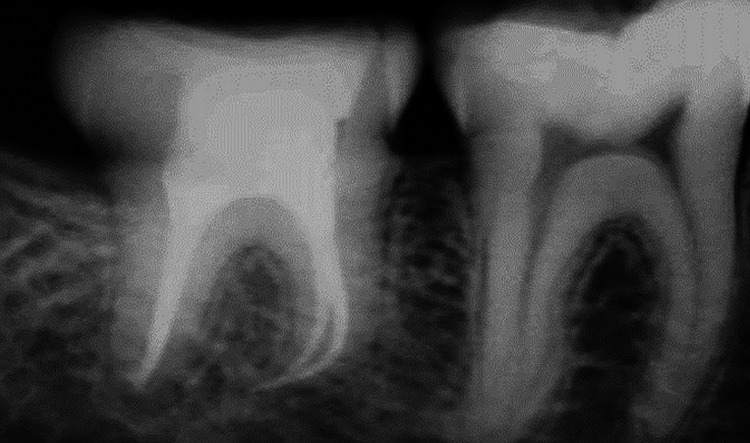
Radiographic evaluation of the 48 tooth after transplantation

Analgesics (paracetamol 500 mg) and antibiotics (amoxicillin 500 mg t.i.d. (three times a day), Metrogyl 400 mg t.i.d.) were administered orally to all patients. Advice on maintaining good dental hygiene and a liquid-only diet was advised for the first two weeks. After a week, the sutures and wire were taken out. After the third molars were transplanted, they were held in place with a wire splint or silk suture for two to three weeks. Closed apex molars were treated regularly with root canal therapy after one month. All the cases were evaluated for pain, infection, mobility, ankylosis, and resorption at postoperative two weeks, one month, three months, and six months. The 47 tooth was extracted in the figure given below and replaced with the 48 tooth (Figure 3).

The severity of the pain was measured using a visual analog scale (VAS) with ratings of 0 (no pain), 1 (very little pain), 2 (some discomfort), 3 (moderate pain), and 4 (intense pain). The presence of infection was assessed based on the presence of purulent discharge and recorded as present-p or absent-a. The degree of tooth mobility was clinically assessed by holding the tooth firmly between the handles of two metallic instruments or by using one metallic instrument and one finger. The radiographs of a patient with ankylosis revealed a lack of the lamina dura and a closure of the PDL gap. Both present-p and absent-a notations were made. A radiographic examination of the root surface revealed radiolucency, indicative of resorption. Both present-p and absent-a notations were made. Success evaluation criteria are commonly utilized in published works [10-12]. At the conclusion of the observation period, the tooth transplant was considered successful if it met the following conditions: the transplanted tooth was functioning normally without excessive mobility, and physiologic mobility was allowed [10-12]. Radiographically, there is no root resorption and a normal PDL space and the lamina dura [13].

Statistical analysis

The readings were tabulated and statistically analyzed by IBM SPSS Statistics for Windows, Version 22 (Released 2013; IBM Corp., Armonk, New York, United States). The pain was assessed by Friedman and Wilcoxon signed-rank tests. Mobility, ankylosis, and resorption were assessed by the chi-square test.

## Results

The descriptive statistics for pain are shown in Table 1.

**Table 1:** Descriptive statistics for pain

Days	Mean	Standard deviation
2 weeks	2.45	0.51
1 month	1.80	0.62
3 months	0.80	0.89
6 months	0	0

The average pain score was 2.72 at two weeks and 0 at six months, with pain being at its highest and lowest points during those times (Table 2).

**Table 2:** Mean score for pain

Time	Mean pain score
2 weeks	2.72
1 month	2.05
3 months	1.22
6 months	0

A p-value less than 0.05 for the Friedman test indicates that there was significant difference in VAS scores at different time intervals (Table 3).

**Table 3:** Friedman test results for pain readings N: number of variables; Df: degree of freedom

Test variables	Values
N	20
Chi-square	28.667
Df	2
p-value	0.001

The Wilcoxon signed-rank test results confirmed that the pain was highly statistically significant at different time intervals (Table 4).

**Table 4:** Wilcoxon signed-rank test results for pain

Tests value	1 month-2 weeks	3 months-2 weeks	3 months-1 month
Z	-3.127	-3.835	-3.245
p-value	0.002	0.000	0.001

Infection was not seen in any case at all times. There was absence of purulent discharge at all time intervals. Mobility was highest at two weeks and gradually reduced by three months to six months. Two cases reported mobility that was not statistically significant. The chi-square test results show that there is significant correlation between the time and response (Table 5).

**Table 5:** Chi-square test results for mobility Df: degree of freedom

Tests	Value	Df	p-value
Pearson's chi-square	95.434	8	0.001
Likelihood ratio	117.288	8	0.001
Linear-by-linear association	48.312	1	0.001

The presence of ankylosis was seen in only one patient. The chi-square test showed that there is no correlation between the time and response (Table 6).

**Table 6:** Chi-square test results for ankylosis Df: degree of freedom

Tests	Value	Df	p-value
Pearson's chi-square	0.000	3	1.000
Likelihood ratio	0.000	3	1.000

After six months, there was resorption in two cases, which needed to be extracted. The chi-square test results show that there is no correlation between time and response (Table 7).

**Table 7:** Chi-square test results for resorption Df: degree of freedom

Test	Value	Df	p-value
Pearson's chi-square	4.029	3	0.258

Success rate

Out of the 20 cases, four patients required extractions due to resorption and grade 3 mobility. One patient showed ankylosis. The success rate of the autotransplantation was 75%. Fifteen patients showed well-defined lamina dura without ankylosis and resorption.

## Discussion

By performing an autotransplant, dentists have a shot at perfectly recreating the patient's original anatomy and function. In the upper incisor area, Kristerson and Lagerstrom [17] reported a success rate of 82% for transplanting, while Kugelberg et al. [18] reported that in the maxillary incisor area, 96% of immature premolars and 82% of mature premolars were successful four years following surgery. Adolescents may benefit from having premolars transplanted to replace lost maxillary incisors, according to the findings of the research by Czochrowska et al. [19].

In this research, 75% of the patients who had an autogenous third molar transplant had successful outcomes. These findings run counter to those published by Watanabe et al. [20], Kallu et al. (68%) [21], and Huth et al. (74%) [22] counter to those published by Watanabe et al. [20], Kallu et al. (68%) [21], and Huth et al. (74%) [22]. According to Park et al. [23], preserving PDL requires a gentle and nontraumatic evacuation of the donor's teeth. To prevent this, one oral surgeon followed a standard operating procedure for all transplants in the current research. The iatrogenic damage of a donor tooth PDL may be greatly reduced and the success rate of surgery improved, according to some research, if a copy of the donor's tooth is used to form the recipient location before the tooth is extracted [24]. The donor’s tooth and the receiver’s location are matched in this research. The third molar could not be placed into the recipient site right away in a few patients, despite a careful preparation of the socket. This occurred because the mesiodistal dimension of the donor's tooth was somewhat smaller than the mesiodistal size of the recipient site. Tsukiboshi [1] proposed that, in such a situation, the donor’s tooth may be ground down to a proximal height of 0.5 mm. This procedure lengthens the amount of time spent outside of the mouth and necessitates a careful management of the donor’s tooth, which raises the risk of PDL damage. This explains why it was the only factor shown to alter prognosis in the research by a statistically meaningful amount.

The kind of fixation and its impact on periodontal recovery is still up for debate. According to Mendes and Rocha, splints might have an impact on oral hygiene and cause issues, such as inflammatory root resorption and ankylosis. As a consequence, the procedure's efficacy over the long run may be diminished. According to a summary of stabilization methods by Armstrong et al., short-term flexible splinting is preferable. Suture stabilization of the donor's tooth or no stabilization at all was offered, with similar high success rates (over 81.4%) [25]. In this particular study, sutures were used to hold the tooth in place. In the current investigation, all excised third molars were fully formed teeth; therefore, revascularization of the pulp after transplantation and the necessary root canal treatment was very improbable. It has been claimed that early endodontic therapy may halt the spread of degradation products and toxins from infected nonvital pulp tissue via the apical foramen, accessory canals, and dentinal tubules. As a result, endodontic therapy has the potential to halt bone loss. According to Andreasen et al. [26], completely rooted premolars that were endodontically treated four weeks after transplanting had a 98% five-year survival rate. Therefore, a root canal treatment was done after one month in this research. The findings demonstrated that prompt autotransplantation of the third molar in the mandible is a viable option for the replacement of a nonrestorable tooth.

The study has certain limitations, such as the limited sample size, and further research over a longer period of time is required to fully understand the findings.

## Conclusions

A dependable method of replacing missing teeth is the third molar autotransplant technique. The surgical procedure must be as painless as possible in order to protect the PDL of the tooth that will be transplanted. The root's level of development is also related to the likelihood of success, with a fully grown root having a worse prognosis. In India, immediate third molar autotransplantation is a feasible and effective treatment option to replace a tooth that cannot be saved.
